# Ferroptosis in sepsis: The mechanism, the role and the therapeutic potential

**DOI:** 10.3389/fimmu.2022.956361

**Published:** 2022-08-05

**Authors:** Lei XL, Zhao GY, Guo R, Cui N

**Affiliations:** Department of Critical Care Medicine, Peking Union Medical College Hospital, Chinese Academy of Medical Science and Peking Union Medical College, Beijing, China

**Keywords:** sepsis, ferroptosis, Nrf2, mTOR, autophagy, P53, organ damage, treatment

## Abstract

Sepsis is a common critical illness in the Intensive care unit(ICU) and its management and treatment has always been a major challenge in critical care medicine. The dysregulated host response to infection, causing systemic multi-organ and multi-system damage is the main pathogenesis. Notably, intense stress during sepsis can lead to metabolic disturbances of ions, lipids and energy in the organism. Ferroptosis is an iron-dependent, non-apoptotic cell death distinguished by a disruption of iron metabolism and iron-dependent accumulation of lipid peroxides. Mounting researches have established that ferroptosis has an essential part in anti-inflammatory and sepsis, and drugs targeting ferroptosis-related molecules, such as ferroptosis inhibitors, are gradually proving their effectiveness in sepsis. This paper summarizes and reviews the pathogenesis of ferroptosis, its regulatory network, and its vital involvement in the initiation of sepsis and related organ damage, and finally discusses the possible target drugs provided by the above mechanisms, describes the dilemmas as well as the outlook, in the hope of finding more links between ferroptosis and sepsis and providing new perspectives for the future treatment of sepsis.

## Introduction

Sepsis has long been a major global health challenge. According to a global burden of disease analysis, sepsis-related deaths were responsible for one-fifth of all deaths in 2017, and the estimated number of sepsis cases and sepsis-related deaths in that year was more than double the previous count, implying that sepsis may be more prevalent and lethal than our current estimates, and that sepsis deserves more research and concerns (1). Advances in the treatment of sepsis are still confined to symptomatic management such as fluid resuscitation and organ support (2). And there is still a gap in effective and available drugs for the management of sepsis, so the search for new directions and available mechanisms is far-reaching. Sepsis 3.0, the latest version of sepsis diagnostic criteria (3), defines sepsis as “life-threatening organ dysfunction caused by a dysregulated response of the body to infection.” and its underpinning circulatory and cellular/metabolic abnormalities contribute to increasing the mortality (4). Sepsis has been described as a failed starvation response, emphasizing that the body experiences an intense starvation response involving the production of high-energy metabolites such as lactate and free fatty acids (5). Exploring the mechanisms associated with these circulatory and metabolic abnormalities, such as oxidative stress, autophagy, and ferroptosis, which are closely related to cellular ion metabolism, lipid peroxidation, etc., and finding potential targets may offer new prospects for the management and treatment of sepsis.

**Figure 1:**
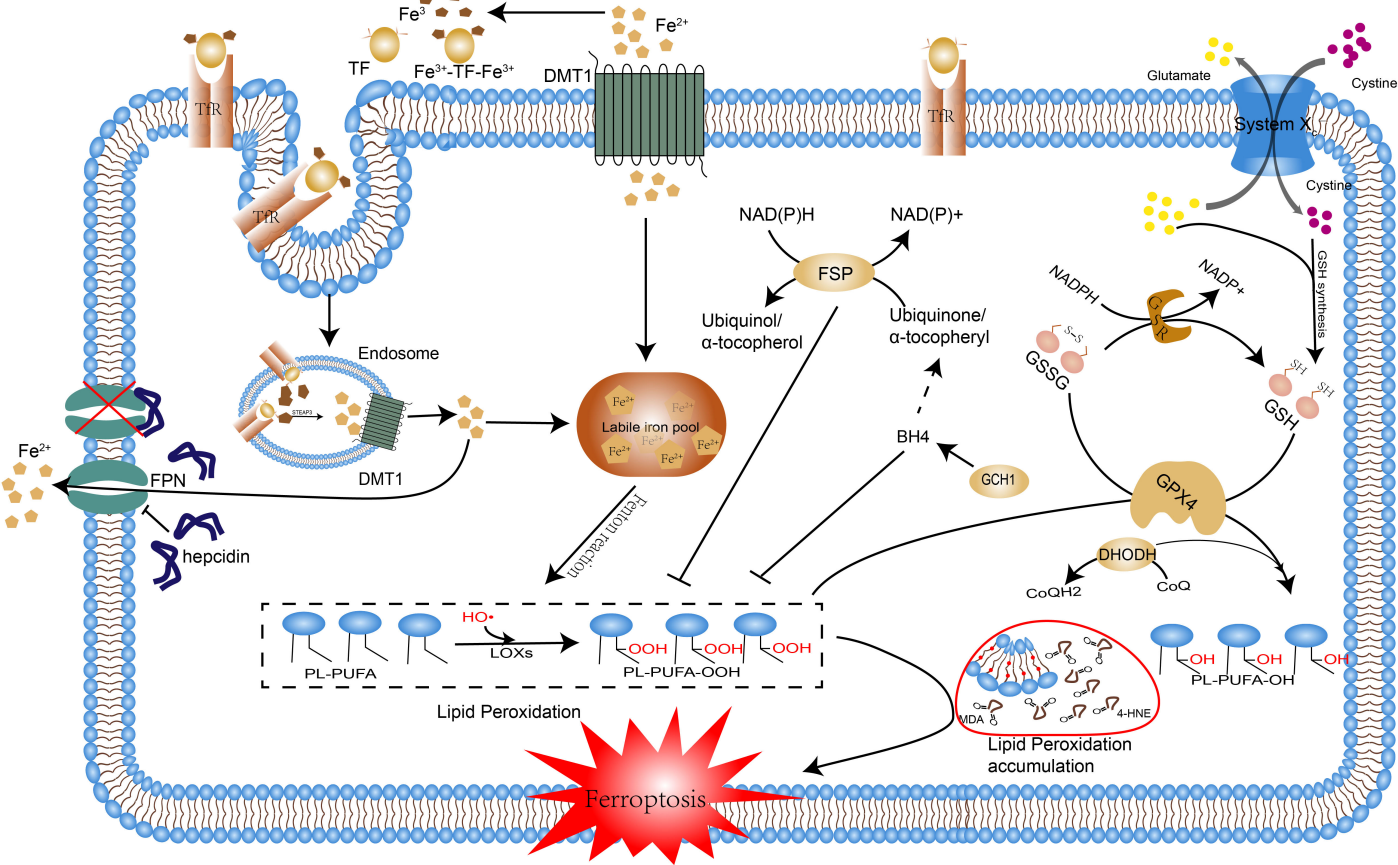
The occurrence mechanism of ferroptosis. The pathways by which ferroptosis is caused are reviewed, including disorders of iron metabolism, lipid peroxidation, and breakdown of the antioxidant system. Excessive accumulation of intracellular iron induces lipid peroxidation through the Fenton reaction, the disruption of the antioxidant system leads to the accumulation of lipid peroxidation products and the above processes ultimately lead to ferroptosis. Abbreviations: Tf, transferrin; TfR1, transferrin receptor 1; Fe2+, ferrous iron; Fe3+, ferric iron; FPN, ferroportin; FT, ferritin; DMT1, divalent metal transporter 1; HO•, hydroxyl radicals; LOXs, lipoxygenases; PL, phospholipid; PLOOH, phospholipid hydroperoxide; PUFA, polyunsaturated fatty acid; MDA, Malonaldehyde; 4-HNE, 4 hydroxynonenal; GPX4, glutathione peroxidase 4; GSH, glutathione; GS-SG, oxidized glutathione; FSP1, ferroptosis suppressor protein 1; CoQ10, coenzyme Q10; DHODH, dihydroor otate dehydrogenase; DHO, dihydroorotate; BH4, Tetrahydrobiopterin; GCH, GTP cyclic hydrolase;.

Ferroptosis is a distinctive form of programmed cell death with unique morphological, genetic, biochemical features, and of which intracellular aberrant iron metabolism and lipid peroxidation are two indispensable and emblematic processes. Ferrous iron interacts with polyunsaturated fatty acids (PUFA) in cell membranes by providing free electrons through the Fenton reaction, producing lipid hydroperoxides (LOOHs), a process known as lipid peroxidation, which can lead to an excessive accumulation of these LOOHs if the antioxidant system is defective, triggering cellular ferroptosis (6). Normally, the oxidative system consisting of iron ion, Fenton reaction and ROS and the antioxidant system consisting of glutathione peroxidase 4(GPX4), glutathione, Xc-system and some newly identified pathways (**Figure 1**) antagonize each other to maintain the proper physiological status of the cells and the organism (7). In settings such as infection, inflammation, and cancer, disruption of this homeostasis can promote ferroptosis, thus resulting in or exacerbating adverse outcomes. In recent years, the contribution of ferroptosis to the development of various diseases has been extensively discussed. Especially in the oncological context, the role of ferroptosis in the development, treatment, and drug resistance of hepatocellular carcinoma, gastric cancer, and breast cancer has been demonstrated (8). In viral infections, increased iron in cells and intestinal iron uptake can lead to ferroptosis (9). The tissue damages caused by bacterial infections are also associated with the activation of ferroptosis, e.g. Pseudomonas aeruginosa and Mycobacterium tuberculosis can cause lung tissue damage through ferroptosis by inducing lipid peroxidation (10, 11). Bacteria require iron for reproduction and cellular ferroptosis releases excessive intracellular iron for their use, therefore, ferroptosis may be a feast for bacterial infections, which in turn can provide raw materials for lipid peroxidation such as fatty acids and reactive oxygen species(ROS), thereby circulating and exacerbating the infection, which may eventually proceed to sepsis and cause multi-organ failure. Notably, immune cells such as macrophages, T cells, and B cells themselves undergo ferroptosis resulting in reduced numbers and function, and ferroptosis-dead cells, in turn, can be identified by immune cells and thus trigger a series of inflammatory or specific immune responses (12).Overall, ferroptosis promotes infection by providing the raw material for bacterial multiplication and reduces the body’s immune function or even induces autoimmunity leading to immune dysfunction, which are undoubtedly crucial contributing factors to the development of sepsis.

Whether the above ideas about the involvement of ferroptosis in sepsis can be confirmed and whether the exploration of ferroptosis as a direction of intervention can bring a breakthrough in the treatment and management of sepsis are yet to be investigated in depth. This review will summarize the progress of research on ferroptosis in sepsis and its associated organ damage. And the role of ferroptosis may also be different for sepsis occurring in specific conditions, we also followed and discussed the literature representing contrasting views, hoping to be useful for further studies.

## The mechanism of ferroptosis

### Iron metabolism imbalance

As an essential cofactor, iron participates in various physiological and biochemical reactions, most notably redox reactions. Pathological iron overload in tissues can promote the production of ROS, which can trigger oxidative stress, ferroptosis, and other toxic reactions (13). Usually, iron metabolism in the body consists of uptake, activation, storage, and recycling processes, and the corresponding regulatory components need to interact with each other to ensure that the iron cycle is running properly. Dietary intake and erythrocyte degradation by macrophages represent the predominant extrinsic source of iron and recycling pathway, respectively; the iron-responsive element/Iron regulatory proteins(IRE/IRPs) mechanism controlling intracellular iron levels through post-transcriptional regulation, and the hepcidin- ferroportin-1 (Fpn1) axis modulating cellular iron transfer and uptake (14).

Iron flows and circulates in the form of Fe3+ and Fe2+. Cellular iron is mainly absorbed into the cell as trivalent iron and Fe3+ combines with transferrin (TF) in serum to form a complex, which is recognized by transferrin receptor 1 (TfR1) on the cell membrane and transported into the cell *via* endonucleosomes formed by endocytosis (15). Intracellularly, Fe3+ in the endosome is converted to Fe2+ by the high iron reductase six-transmembrane epithelial antigen of the prostate (STEAP3). Free Fe2+ can be transported from the endosome to the cytoplasm *via* the divalent metal transporter 1 (DMT1), then Fe2+ enters the metabolically active pool, the labile iron pool (LIP), to contribute to metabolic activities. Excess intracellular iron stores as ferritin or exits the cell *via* the iron transporter protein FPN1, the only iron export protein identified at present, and its significance in intestinal iron absorption and intracellular iron export is well confirmed by the lethal effect of complete or selective inactivation of the FPN1 gene in the embryo (16). Hepcidin is a cysteine-rich antibacterial peptide synthesized and secreted by the liver, which negatively regulates iron absorption and export by binding to iron transport protein FPN1 and facilitating its internalization and degradation. The hepcidin-ferroportin-1 (Fpn1) axis, which reduces plasma iron concentrations in response to infection and inflammation, limits the availability of iron to invading microorganisms (17). IRPs regulate iron homeostasis by binding to IRE region of particular protein mRNAs and stabilizing or degrading them to regulate the translation of related proteins. When intracellular iron decreases, IRPs bind to mRNAs encoding TFR and ferritin to promote TfR synthesis and specifically inhibit ferritin translation, a process that promotes intracellular iron uptake and inhibits its storage, increasing intracellular iron availability. When intracellular iron is overloaded, this process is restrained (18). In addition to TfR and ferritin, IRE/IRP is also engaged in the transcriptional modulation of other iron metabolism-related proteins such as erythroid 5-aminolevulinate synthase (ALAS2), DMT1, and FPN1 (19, 20). As a general rule, the above regulatory mechanisms work synergistically to ensure the conversion, recycling, and utilization of Fe3+ and Fe2+ in the body. In the presence of infections, inflammation, tumors, drugs and other stimuli, aberrations in the above iron metabolic processes or their regulatory systems can lead to an impairment of intracellular iron levels, and it is the pathological accumulation of iron that is the key step in the development of ferroptosis. Pseudolaric acid B induces ferroptosis by upregulating the transferrin receptor and thereby increasing intracellular iron levels (21).In addition, the lipogenic regulator sterol regulatory element-binding protein 2(SREBP2) can directly invoke the transcription of transferrin (TF), thereby reducing the intracellular iron pool and enhancing the resistance of cells to ferroptosis inducers (22). In lung cancer, knockdown of ubiquitin‐specific proteases(USP35) promotes FPN1 ubiquitination and degradation, and FPN-dependent cellular iron exclusion is reduced, thereby triggering iron overload and ferroptosis in lung cancer cells (23). the protective function of FPN1 in ferroptosis of human myeloid cells has been experimentally confirmed and is proposed to be a novel therapeutic target (24). It has been shown that several molecules can simultaneously affect several of these regulatory mechanisms, disrupting iron homeostasis and inducing the onset of ferroptosis. Dihydroartemisinin (DAT) triggers lysosome-mediated degradation of autophagy-independent ferritin and increases cellular free iron levels. Also, by binding to cellular free iron and thus stimulating the binding of IRPs to mRNA molecules containing IRE sequences, it affects IRP/IRE-controlled iron homeostasis, thereby further increasing cellular free iron and increasing cellular susceptibility to ferroptosis (25). Free iron is associated with iron-dependent lipid autoxidation/peroxidation through Fenton reactions or enzymatic catalysis on the one hand. On the other hand, it is involved as a cofactor in some oxidoreductases or lipid metabolizing enzymes, such as lipoxygenase, which catalyze lipid oxidation and protein metabolism, all of which can contribute to the occurrence of ferroptosis. This shows that there are also many interactions between the two prominent features of ferroptosis, iron overload, and lipid peroxidation.

### Lipid metabolism

Lipid peroxidation, a crucial step and feature of the onset of ferroptosis, is mainly driven by the peroxidation of free polyunsaturated fatty acids (PUFAs) in the presence of lipoxygenase and ROS. Firstly, PUFAs such as arachidonic acid (AA) and adrenoyl (AdA) are sequentially synthesized into lipids and inserted into membrane phospholipids to form PUFA-PL complexes, catalyzed by Acyl-CoA synthetase long-chain family member 4 (ACSL4) and lysophosphatidylcholine acyltransferase 3 (LPCAT3). Subsequently, it is oxidized to lipid hydroperoxides by the action of peroxidases (LOXs), and then ferroptosis is activated (26). Targeting the above key enzymes to inhibit the occurrence of ferroptosis has become an emerging therapeutic approach for a variety of diseases. Thiazolidinedione hypoglycemic agents and safranin have been shown to effectively inhibit ferroptosis by inhibiting ACSL4 (27, 28).

The metabolic processes of fatty acid synthesis, storage, catabolism, and lipophagy affect the type and concentration of intracellular lipids and determine the susceptibility of cells to ferroptosis, which affects the initiation of it. Under energy stress, activated Adenosine 5’-monophosphate (AMP)-activated protein kinase(AMPK) inhibits ferroptosis by suppressing the biosynthesis of PUFAs and other fatty acids (29). Fatty acids are categorized into monounsaturated fatty acids (MUFAs) and polyunsaturated fatty acids (PUFAs), the synthesis of which is competitive and can also interact directly with each other. Magtanong et al. found that exogenous MUFAs were able to inhibit lipid accumulation at the plasma membrane *via* an ACSL3-dependent pathway and remove PUFAs from that location in the cell (30). Thus, the balance between MUFAs and PUFAs needs to be finely adjusted to control the onset of ferroptosis. Whether the interaction of other lipids such as saturated fatty acids and phospholipids with PUFAs affects the occurrence of ferroptosis needs to be further investigated. The autophagic degradation of lipid droplets, known as lipophagy, is a major mechanism of lipid transformation in many cells, and lipophagy-derived fatty acids can play a complementary role and promote RSL3-induced ferroptosis in hepatocytes (31).In addition, either enhancing Tumor protein D52(TPD52)-dependent lipid storage or inhibiting autophagy-related genes(ATG5)- and RAB7A (a member of the RAS oncogene family) -dependent lipid degradation could prevent RSL3-induced lipid peroxidation and ferroptosis. Lipophagy promotes lipid peroxidation in ferroptosis by reducing lipid storage and increasing lipid degradation (32). Lipid peroxidation is a dynamic and complex process, either by intervening in the synthesis and storage of oxidative substrates or by influencing the activity of key enzymes, which can further control the onset of ferroptosis and offer new hope for the treatment of many diseases such as tumors, infections, and immunological disorders.

### Antioxidant systems

#### Xc–GSH-GPX4 network

The Xc–GSH-GPX4 network is generally considered to be the main antioxidant roadblock against ferroptosis. The Xc-system, also known as the Cystine/Glutamate reverse transporter, consists of the light chain solute carrier family 7 member 11(SLC7A11) and the heavy chain solute carrier family 3 member 2 (SLC3A2) (33).Glutathione (GSH) is the most vital antioxidant, maintaining the balance of redox reactions. Glutathione peroxidase 4 (GPX4) is a notable member of the selenoprotein family, whose main role is to reduce peroxides (e.g. R-OOH) to the corresponding alcohols (R-OH). This antioxidant system is used to combat ferroptosis by transporting cystine through the Xc- system to synthesize glutathione (GSH), which in turn assists GPX4 in the reduction of peroxides (34).

The modulation of ferroptosis through upregulation or inhibition of these antioxidant systems can influence the development or outcome of a variety of diseases. Various drugs and molecules have been found to induce ferroptosis by inhibiting the activity of the Xc- system. Erastin is a classical inhibitor of the Xc- system and a common agent used in the construction of ferroptosis models, while ATF3 has a synergistic effect on erastin (35). Glutathione is made from cysteine and is catalyzed by glutamate-cysteine ligase and glutathione synthase. There is no doubt that the depletion of glutathione or the lack of cysteine, the raw material for its synthesis, can cause ferroptosis to occur (36, 37). GPX4 activity and synthesis are in turn associated with selenium and GSH. Selenium upregulates GPX4 at the transcriptional level by driving the transcriptional activators transcription factor AP-2T(FAP2c) and Sp1 (38). Selenium supplementation has been shown to enhance the expression of GPX4 in T cells, protect them from ferroptosis, thereby increasing the number of helper T cells, and promote antibody responses in immunized mice and young adults humans (39). GPX4 consumes two molecules of GSH to produce one molecule of GSSG, thereby preventing lipid peroxidation. The effect of GPX4 can be facilitated by molecules or drugs that recruit GSH *in vivo*, such as nicotinamide mononucleotides (NMN), which recruit GSH to enhance resistance to GPX4-mediated ferroptosis and thus reduce UV-related skin damage (34). Conversely, the GPX4 inhibitors RSL3 and ML210/162 can cause ferroptosis in resistant tumor cell lines and are lethal to residual tumor cells (40). At the genetic level, GPX4 knockout mice develop renal failure due to the loss of GPX4’s inhibitory effect on ferroptosis (41).

As the principal antioxidant component, GSH is involved in a variety of redox reactions in the body, maintaining normal physiological homeostasis. GPX4 is the most critical regulator of ferroptosis and determines the fate of cells. The complementary interplay of the members of the Xc–GSH-GPX4 system is the gatekeeper that ensures that ferroptosis occurs in the right environment, is regulated at the right time, and contributes to disease treatment.

#### CoQ10-FSP1-NADH axis

From the time ferroptosis was defined, GPX4 has been treated as its most prominent inhibitory molecule. As research progressed, it became evident that inhibition of GPX4 activity did not necessarily trigger ferroptosis. Almost concurrently, Bersuker and Doll et al. used a gene scan technique (CRISPR/Cas9s) to find that ferroptosis-suppressor-protein 1(FSP1) complemented the inactivation of GPX4 in osteosarcoma cells and MCF7 ferroptosis resistant cell lines, respectively, thus suggesting that FSP1 is a potent inhibitor of ferroptosis. FSP1 on the plasma membrane can prevent lipid peroxidation by reducing ubiquinone (oxidized CoQ10) with NADPH as a cofactor. This process is neither dependent on GPX4 nor requires GSH as a cofactor (42, 43).Thus, FSP1 may be a ferroptosis suppressor independent of GPX4, and the expression level of FSP1 determines the sensitivity of cells to ferroptosis. FSP1-related molecules or inhibitors are also being investigated, such as plasma-activated medium (PAM), which reduces the expression of FSP1 and causes ferroptosis in lung cancer (44). Pharmacological research has found that the small molecule NPD4928 can directly bind to FSP1 to inhibit its activity and promote ferroptosis (45).

The discovery of the CoQ10-FSP1-NADH axis proves that GPX4 is not the only inhibitor of ferroptosis, FSP1 is an inhibitor that has its effects through GPX4. Further mechanisms whereby ferroptosis can be inhibited continue to be identified.

#### DHODH-CoQH2 axis

Dihydroorotic dehydrogenase (DHODH) is the fourth rate-limiting step in the *de novo* biosynthesis of pyrimidines and is localized in the inner mitochondrial membrane. It catalyzes the oxidation of dihydroorotate acid (DHO) to orotate (OA) and the reduction of CoQ (ubiquitin) to CoQH2 (ubiquinone), which links to the respiratory complex and participates in the transfer of electrons in the oxidative respiratory chain (46). Considering its role in redox reactions, Mao and his team found that the substrates and products of DHODH, namely dihydroorotate acid (DHO) and orotate (OA), had contrasting effects on the onset of ferroptosis utilizing global metabolomic analysis. Follow-up studies revealed that ferroptosis occurred in GPX4 low expressing cells after DHODH was inhibited, while GPX4 high expressing cells showed increased sensitivity to ferroptosis. The authors also explored the interaction between DHODH and the two classical inhibitory mechanisms known for ferroptosis and confirmed that DHODH does not regulate the expression levels of other ferroptosis-related molecules such as GPX4, FSP1, and the Xc- system, but can synergize with GPX4 to inhibit mitochondria-related ferroptosis, while acting independently of FSP1 (47). Current studies have shown that SA771726 (Leflunomide metabolite) and Brequinar sodium (BQR), inhibitors of DHODH, play a predominant role in the treatment of tumors such as melanoma, osteosarcoma, breast cancer, etc (48, 49). Whether ferroptosis is involved in this process and whether DHODH inhibitors in combination with GPX4 inhibitors or other ferroptosis inducers can achieve better therapeutic results remains to be further monitored and explored, but is certainly a direction of great clinical potential.

#### GCH1-BH4 axis

Tetrahydrobiopterin (BH4) is an essential cofactor for a variety of enzymes, and its role in maintaining cellular redox stability is generally recognized, but its extended effects, such as the inhibition of ferroptosis, have only recently been demonstrated. In a study based on genome-wide activity scanning and functional analysis, GTP cyclic hydrolase 1 (GCH11), the rate-limiting enzyme for BH4 synthesis, was identified as a potential inhibitor of ferroptosis. Subsequent studies demonstrated that the GCH1-BH4 axis inhibits ferroptosis by controlling BH4 synthesis, reducing intracellular CoQ and ROS accumulation, and especially protecting phospholipids with two polyunsaturated fatty acid tails (50). Another gene scan based on CRISPR screens also confirmed that GCH1-BH4, a metabolic axis of ferroptosis control, determines the sensitivity of tumor cells to ferroptosis (51). Besides, Hu et al. found that GCH1/BH4 inhibited erastin-induced ferritin toxicity in colorectal cancer by selectively inhibiting nuclear receptor coactivator 4(NCOA4)-mediated ferritin autophagy and affecting iron metabolism (52). This shows that the axis has a direct inhibitory effect on both lipid oxidation and iron metabolism, the two main keys through which ferroptosis occurs. The GCH1-BH4 axis may be an independent inhibitory mechanism from the GSH/GPX4 and FSP1/CoQ inhibitory pathways and is a powerful inhibitor. The application of relevant modulators of this axis deserves to be explored in a variety of pathological states.

## Typical regulatory pathways associated with ferroptosis

### Nrf2

**Figure 2:**
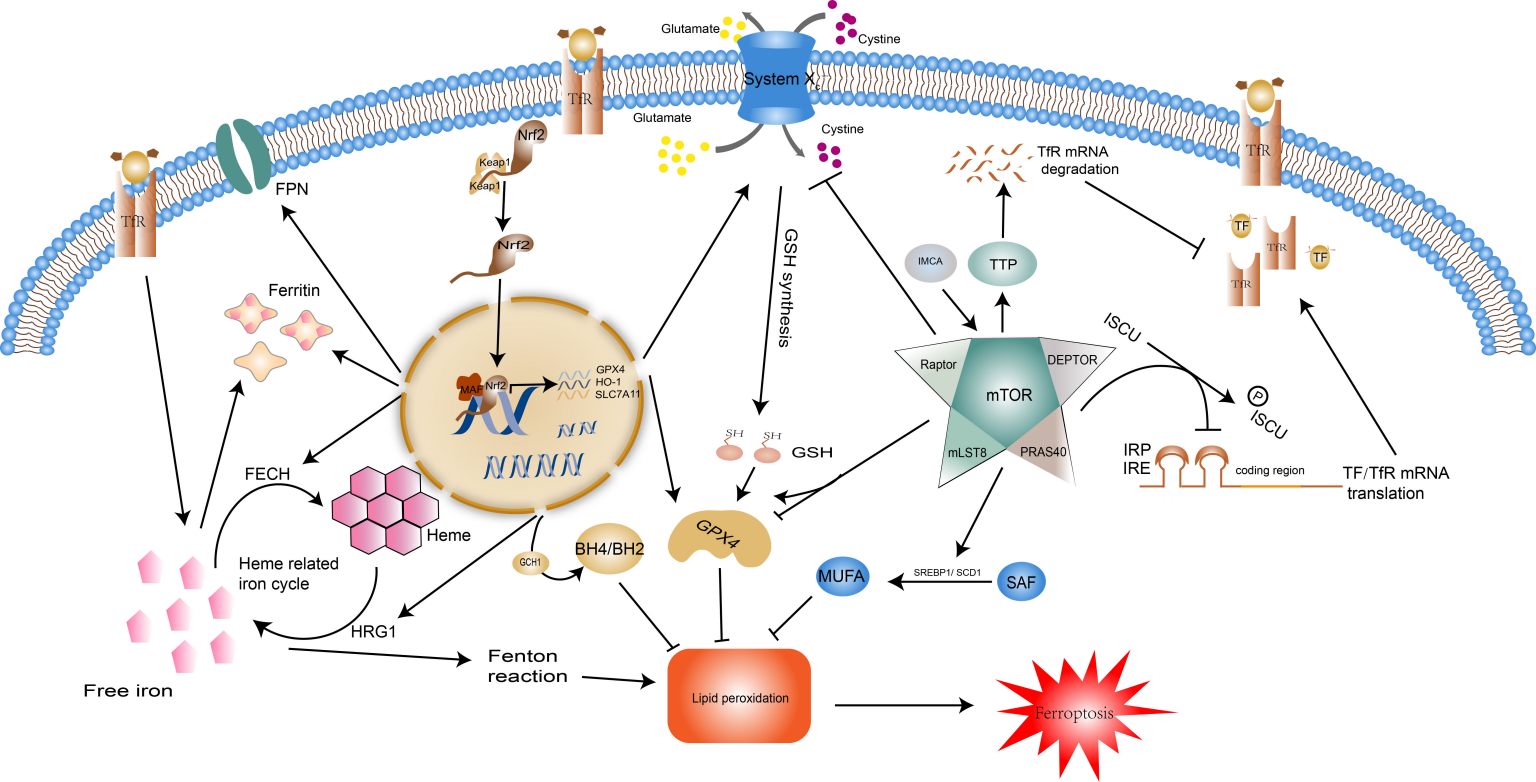
Regulatory pathways of ferroptosis: Nrf2 and mTOR. Normally, Nrf2 is bound by keap1 and then degraded. When the body is under oxidative stress, it translocates into the nucleus and regulates transcription. Nrf2 moderates ferroptosis by acting on iron metabolism and antioxidant systems. Nrf2 can promote the expression of ferritin and FPN, HRG1 and FECH to control the recycling, storage, and utilization of iron. Additionally, the expression of SLC7A11, GPX4, HO-1, and GCH1 facilitated by Nrf2 enhances the effect of the antioxidant system. The impact of mTOR on ferroptosis is two-fold. For one thing, mTOR reduces intracellular iron concentrations by inhibiting TF/TfR transcription and promoting TfR degradation, and enhances the synthesis of monounsaturated fatty acids, two pathways that inhibit the onset of ferroptosis. In contrast, mTOR facilitates ferroptosis by repressing the Xc system and reducing GSH synthesis. mTOR also has a dual effect on GPX4. Abbreviations: Nrf2, nuclear factor erythroid 2-related factor 2; FPN, ferroportin; HRG1, heme responsive gene-1; FECH, ferrochelatase; SLC7A11, solute carrier family 7 member 11; GPX4, glutathione peroxidase 4; HO-1, heme oxygenase-1; GCH1, GTP Cyclohydrolase 1; Tf, transferrin; TfR, transferrin receptor.

The transcription factor nuclear factor erythroid 2-related factor 2 (Nrf2) governs the expression of genes related to oxidative stress and is an integral component in the maintenance of redox stability and resistance to oxidative stress in the body (53). In the regulation of ferroptosis, Nrf2 suppresses lipid peroxidation by regulating the expression of the components of the Xc- system (SLA7C11), GSH synthesis, GPX4 activity, and iron homeostasis, and even increases the activity of the ferroptosis resistant system GCH1-BH4, thereby controlling the occurrence of ferroptosis in general (**Figure 2**). Overexpressed Nrf2 has been demonstrated in cancers such as breast and bladder cancer to directly bind to the promoter of SLA7C11 in the Xc- system, promoting its transcription, accelerating cysteine transport, and enhancing glutathione synthesis (54, 55). Nrf2 reverses drug resistance in lung small cell lung cancer cells by increasing the expression of GPX4 (56). The regulation of iron metabolism by Nrf2 involves the recycling, storage, and utilization of iron. Firstly, Nrf2 regulates the hemoglobin-related iron cycle and is involved in the destruction and resynthesis of hemoglobin through catalytic enzymes such as heme responsive gene-1(HRG1) and ferrochelatase(FECH), i.e. it participates at both the beginning and end of the cycle of iron recycling (57). Secondly, Nrf2 augments the transcription and synthesis of ferritin and FPN1 to increase iron storage and transfer, preventing intracellular iron overload and thus reducing the reactive oxygen species (ROS) (58, 59). In addition, it has been documented that Nrf2 upregulates GCH1 activity, promotes BH4 synthesis, and protects radioactively damaged cells from oxidative damage (60). This effect undoubtedly increased the suppressive effect of the GCH1-BH4 axis on ferroptosis. Overall, Nrf2 is a negative regulator of ferroptosis, it represses the peroxidation system and enhances the ferroptosis defense system, preventing the occurrence of ferroptosis.

### mTOR

Mechanistic target of rapamycin (mTOR) is a serine/threonine kinase that acts as an integrator of cell growth, differentiation, and function to ensure that the body adapts to environmental stressors. Growth factors, amino acids, energy status, and hypoxia determine cell fate by stimulating the mTOR complex to influence cell metabolism, lipid and protein synthesis, and the type of cell death (61). mTOR restrains ferroptosis (**Figure 2**) by modulating downstream SREBP1/Stearoyl-CoA Desaturase-1 (SCD1)-mediated lipid synthesis (62). SCD1 catalyzes the monounsaturated fatty acids (MUFAs) synthesis from saturated fatty acid (SFA) substrates, which inhibit the process of ferroptosis (63). It is thus clear that mTOR can influence the onset of ferroptosis by mediating the accumulation of polyunsaturated fatty acids and can also promote cellular resistance to ferroptosis through monounsaturated fatty acids. mTOR also has regulatory effects on the cellular IRE/IRP and TF and its receptor (TfR). It can inactivate the binding activity of IRP and IRE by consistently increasing the scaffold protein (ISCU) protein levels (64). Tristaprolin (TTP), a downstream target of mTOR, can bind to the TfR1 mRNA and enhances its degradation, thereby affecting iron transfer into the cell (65). This regulation of iron metabolism by mTOR can only be maintained within a moderate range, otherwise, the iron overload will lead to an increase in intracellular labile iron instability which can cause oxidative damage and cell death (e.g. ferroptosis) or cause iron deficiency, leading to hematological disorders such as anemia (66). Existing studies indicate that mTOR can affect the degradation of GPX4 and the level of transcription (67, 68). IMCA induces ferroptosis in colorectal cancer cells by downregulating SLC7A11 expression *via* the AMPK/mTOR pathway, thereby inhibiting cystine transport, reducing GSH synthesis, and promoting reactive oxygen species accumulation (69).These regulatory mechanisms were also validated by Yuchao Gu and colleagues through unbiased proteomic screening of xCT binding chaperones as well as functional validation (70).

### Autophagy

**Figure 3:**
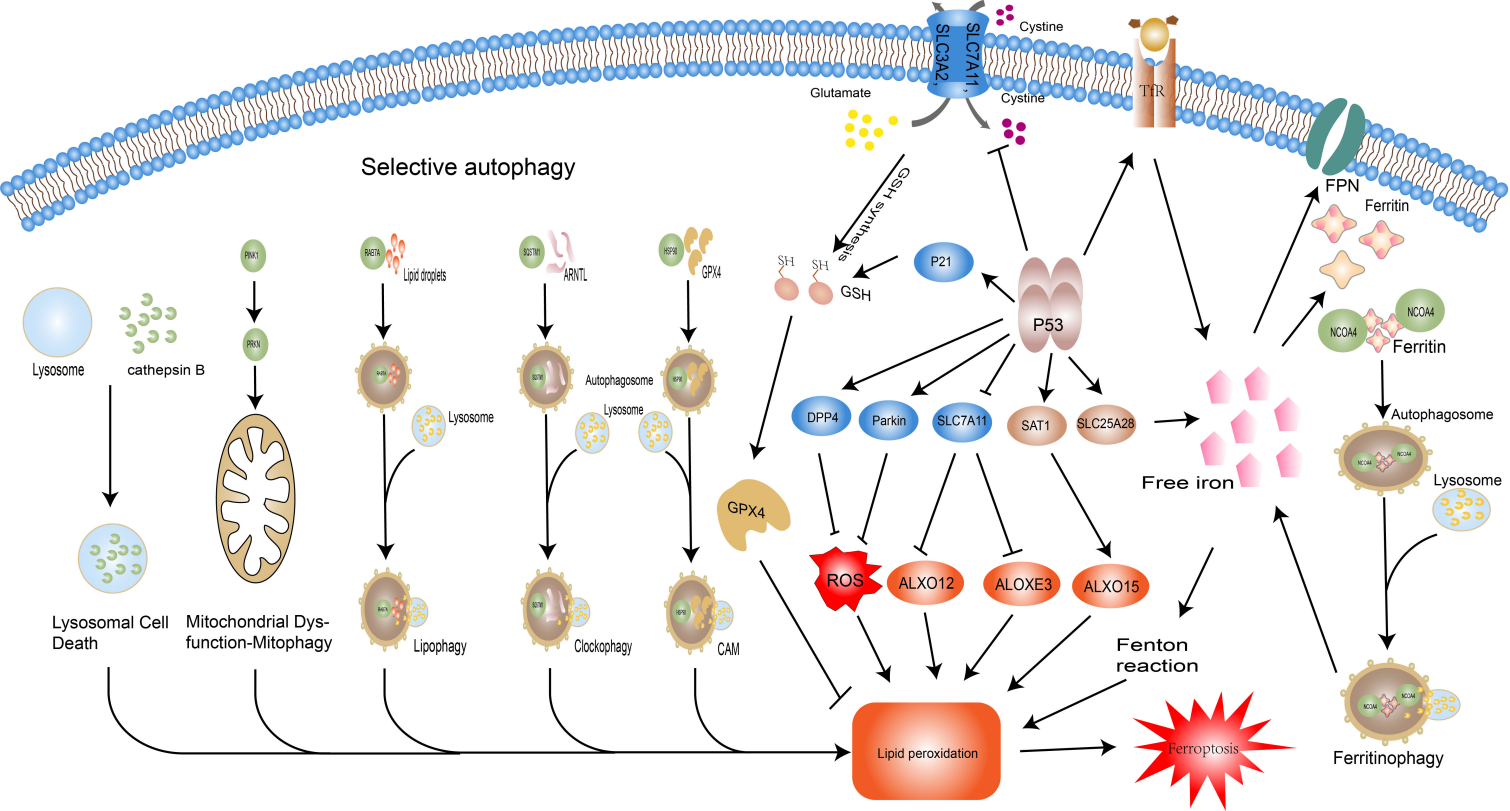
Regulatory pathways of ferroptosis: autophagy and P53.Selective autophagy promotes ferroptosis by facilitating thedegradation of key molecules of it and related organellesautophagy. NCOA4-promoted ferritin degradation; RAB7Amediatedlipid droplets degradation; haperone-mediatedautophagy (CMA) –mediated GPX4 degradation; sequestosome-1(SQSTM1/P62)-mediated Aryl hydrocarbon receptor nucleartranslocator-like protein 1(ARNTL) degradation; overexpressionof cathepsin B significantly promotes ferroptosis via theactivation of lysosomal cell death; PINK1 and PRKN/PARK2regulated mitophagy. The dual role of P53 in ferroptosis isreflected in its regulation of iron metabolism, the GSH-GPX4axis, and lipid metabolism. P53 increases intracellular ironconcentration via SLC25A28 and TfR, thereby promotingferroptosis. P53 inhibits the activity of the Xc system and reducesGSH synthesis, but also slows GSH depletion via P21. In terms oflipid peroxidation, P53 prevents lipid peroxidation through DPP4and Parkin, but also promotes it by manipulating SLC7A11 andSAT1. Abbreviations: NCOA4, nuclear receptor coactivator 4;RAB7A, RAB7A, member RAS oncogene family; CMA, chaperonemediatedautophagy; SQSTM1, sequestosome 1; ARNTL, arylhydrocarbon receptor nuclear translocator like; PINK1, PTENinducedkinase 1; PRKN/PARK2, Parkin RBR E3 ubiquitin proteinligase (PRKN/PARK2); GPX4, glutathione peroxidase 4; GSH,glutathione; DPP4, dipeptidyl peptidase 4; SAT1, spermidine/spermine N1-acetyltransferase 1; SLC7A11, solute carrier family 7member 11; TP53, tumor protein P53.

Autophagy is an invaluable modality in the maintenance of homeostasis, which interacts with a variety of pathological and physiological mechanisms such as protein degradation, oxidative stress, and cell death to counteract stimuli. In particular, selective autophagy takes an integral part in the onset and control of ferroptosis (**Figure 3**). Specific degradation of ferritin, mediated by the selective transport receptor NCOA4, regulates intracellular iron levels. Overexpression of NCOA4 promotes ferritin degradation, adds labile iron, and promotes ferroptosis (71). The closeness of Ferritinophagy and ferroptosis has been reviewed and discussed for its value in the study of metabolic, cardiovascular, and hepatocellular carcinoma cells (72–74), and its potential in sepsis has only been explored in septic myocardial injury (75), and its role in other organ injuries remains to be explored. Analogous selective autophagy relevant to ferroptosis includes RAB7A-mediated lipophagy, i.e. the autophagic degradation of intracellular lipid droplets, molecular chaperone-mediated autophagy (CMA) of GPX4, and sequestosome-1(SQSTM1/P62)-mediated clockophagy, i.e. the selective degradation of the core circadian protein Aryl hydrocarbon receptor nuclear translocator-like protein 1(ARNTL) promoting lipid peroxidation by blocking hypoxia inducible factor 1 alpha (HIF1A)-dependent fatty acid uptake and lipid storage (32, 76, 77). Besides targeting ferroptosis-related molecules, autophagy can also indirectly control the occurrence of ferroptosis through the selective degradation of some organelles. Mitochondrial dysfunction plays a vital role in intracellular iron metabolism disorder and accumulation of ROS, thus providing materials for ferroptosis (78). In contrast, mitophagy can selectively remove senescent or damaged mitochondria to prevent intracellular redox imbalance (79). That is, mitophagy may influence ferroptosis by ensuring the proper functioning of intracellular mitochondria. However, it has also been shown that mitophagy itself can also lead to elevated ROS and trigger ferroptosis (80). The same action may also be played by autophagy involved lysosomal cell death (81), but selective autophagy of these organelles under different stimulation conditions may play distinct effects, the specific regulatory mechanisms, controllable and exploitable targets need to be further explored. The selective autophagy targets the various steps in the onset of ferroptosis, and by affecting intracellular iron levels, lipid peroxidation etc, may achieve precise regulation of ferroptosis, providing a therapeutic direction for a variety of diseases such as tumors and inflammatory conditions.

### P53

The oncogene TP53 (i.e. P53) can be activated under a variety of stress conditions to perform its classical functions such as induction of cell cycle arrest, senescence, apoptosis, maintenance of genomic stability, and regulation of metabolism. Mutations in P53 and its associated signaling pathway inactivation are key events in organismal tumorigenesis (82). Recent studies have shown that mutated P533KR polypeptide loses the three classical functions of P53, namely cell cycle arrest, senescence, and apoptosis, but still exerts oncogenic effects, implying that there are more potential regulatory mechanisms of P53 to be discovered. Thus, ferroptosis was shown to be an emerging regulatory target of P53 (83). With variations in cell type, cellular environment (e.g. stress, under ferroptosis inducers), the effects of P53 on ferroptosis display two faces (**Figure 3**). On one hand, P53 promotes the onset of ferroptosis through the following mechanisms. i. P53 promotes lipid peroxidation by binding to the promoter of SLC7A11 and directly reducing its expression level, thereby affecting the function of GPX4 and relieving its inhibition of ALXO12 and ALOXE3 (two lipoxygenases) activities (83–85). II. P53 promotes the expression of TfR1 and SLC25A28, resulting in abnormal accumulation of reactive iron and increasing cellular susceptibility to ferroptosis (86, 87). III. P53 enhances the transcriptional level of spermidine/spermine N1-acetyltransferase 1 (SAT1) and initiates the involvement of ALOX15 (lipoxygenase) in ROS-induced lipid peroxidation (88). On the other hand, P53 strengthens the anti-ferroptosis system by delaying glutathione consumption and promoting its synthesis through P21, suppressing reactive oxygen species (ROS) by affecting energy metabolism through Parkin, and suppressing NOX1 (NADPH oxidase) activity by repressing Dipeptidyl peptidase-4(DPP4) levels, thereby inhibiting ferroptosis (89–92). P53 has a complex and extensive grip on ferroptosis through the regulation of lipid, energy, and iron metabolism, and the exploitation of this control may open up emerging therapeutic areas for a variety of diseases.

### Other regulatory routes

In parallel to the classical regulatory pathways that have been extensively investigated above, the occurrence of ferroptosis is linked to a variety of other molecules such as AMPK, Ras, etc. to ensure that it occurs at the right time to contribute to the maintenance of organismal stability or disease treatment. AMPK phosphorylated BECN1(beclin1) can directly bind SLC7A11, repress the Xc- system, prevent GSH synthesis and promote lipid peroxidation (93, 94). Both *in vivo* and ex vivo experiments in leukaemia cells have shown that high mobility group box-1(HMGB1) is a key regulatory molecule in erasure-induced ferroptosis (95). Dexazoxane can exert a protective effect against ferroptosis-associated cardiomyopathy by modulating HMGB1 (96). Therefore, an in-depth study on these regulatory networks would be beneficial to understand ferroptosis and that may also provide new therapeutic targets. Other molecules such as signal transducer and activator of transcription 3 (STAT3), FA complementation group D2(FANCD2), heat-shock proteins (HSPs), and other related regulatory networks are also being enriched (97–99).

The regulatory network of the above classical molecules for ferroptosis-related molecules was gradually improved, and possible drugs were explored and experimented. However, problems such as cellular insensitivity and resistance have emerged. Therefore, studies of regulation at the post-transcriptional level have attracted interest, especially for non-coding RNAs, which have made considerable progress. In response to the problem of anti-PD-1 immunotherapy resistance in melanoma, miR-21-3p promotes ferroptosis by enhancing lipid peroxidation, thus providing a new line of hope (100). Under hypoxic conditions, tumors are usually resistant to anticancer therapy, and it was shown that lncRNA-CBSLR induced under such conditions is involved in this resistance precisely by reducing the occurrence of ferroptosis (101). In addition, the role of CircRNA on ferroptosis is also being studied in-depth (102). And besides tumor resistance, the role of non-coding RNA in tumorigenesis, invasion, and metastasis should not be underestimated, as for the role in other diseases, it is yet to be studied further. The more precise the studies targeting the regulation of ferroptosis, the greater the role that can be played in its control and use.

## Ferroptosis in sepsis

### Ferroptosis in the development of sepsis

Sepsis is a lethal disease caused by a dysregulated response to infection resulting in multiple organ damage or failure. The innate immune response, inflammatory cascade, procoagulant, and antifibrinolytic pathways, cellular metabolism, altered signaling, and acquired immune dysfunction are the main pathophysiological alterations in the pathogenesis of sepsis (103). Considerable research has demonstrated that ferroptosis occurs mainly under conditions such as metabolic disorders and oxidative stress in the body and that ferroptosis of immune cells or other cells in which ferroptosis affects the immune response of the body and therefore has a role in the development and progression of sepsis.

Bacterial infection is the main cause of sepsis. During the bacterial infection phase, in patients with severe pancreatitis, ferroptosis of intestinal epithelial cells can disrupt the intestinal barrier and promote the entry of noxious intestinal bacteria and toxins into the circulation and extraintestinal tissues (104). In respiratory infections, Pseudomonas aeruginosa uses host polyunsaturated phosphatidylethanolamine to cause ferroptosis in the bronchial epithelium and promote bacterial invasion (10). During the early stages of the body’s immune response, iron and lipid peroxidation in macrophages increase dramatically, and ferroptosis inducers (RSL3, salazosulfapyridine, and acetaminophen) promote bacterial killing by macrophages (105). However, the dead macrophages caused by mycobacterium tuberculosis exhibited features of ferroptosis such as reduced GSH, iron overload, and lipid peroxidation, and subsequent experiments in mice also demonstrated that this process was associated with the downregulation of GPX4 expression and the onset of ferroptosis (11). T cell lipid peroxidation induces ferroptosis and reduces immunity to infection, and Gpx4 is critical for the homeostatic survival of CD8 T cells and the expansion of CD4+ and CD8+ T cells in response to infection by preventing membrane lipid peroxidation and ferroptosis upon TCR triggering (106). Ferroptosis can be a double-edged sword in sepsis, promoting bacterial invasion, inducing sepsis, and causing the death of immune cells to reduce the body’s immune function, but it can also help immune cells to destroy pathogens. It is therefore extremely important to find a balance between these two effects so that they work positively.

### Ferroptosis in sepsis-related organ damage

#### Sepsis-related cardiac injury (Myocardial injury/new-onset atrial fibrillation)

Cardiac failure due to sepsis often referred to as sepsis-induced cardiomyopathy, is a pathophysiological process that includes increased circulating myocardial inhibitors, oxidative and nitrosative stress, and mitochondrial dysfunction, and is a major cause of deterioration and poor outcomes in patients with sepsis (107). ferroptosis has been investigated in septic cardiomyopathy. Activation of ferroptosis in cardiomyocytes was observed in septic mice and H9c2 myofibroblasts stimulated by Lipopolysaccharide(LPS), and pharmacological inhibition of ferroptosis significantly attenuated cardiac injury, inflammation and improved survival time in LPS mice (75). Dexmedetomidine and Ferrostatin-1 (an ferroptosis inhibitor) have all been proven to alleviate sepsis-related cardiac injury by inhibiting ferroptosis (108, 109). In addition, in a mouse model of LPS-induced endotoxemia, ferroptosis was observed to cause new-onset atrial fibrillation by increasing atrial vulnerability while FPN knockdown or ferroptosis inhibitors slowed the onset of atrial fibrillation (110). It is thus evident that in sepsis, ferroptosis of cardiomyocytes occurs not only affecting the pumping function of the cardiac muscle but also potentially disrupting the cardiac rhythm, further aggravating the damage to cardiac function and leading to circulatory failure.

#### Acute lung injury/ARDS

Acute lung injury (ALI) is a common complication of sepsis. The development of effective drugs and ventilation strategies for sepsis-induced ALI/ARDS has not progressed significantly over the last few decades, and research into the mechanisms involved has become necessary (111). Ferroptosis has been validated to play an influential part in LPS-induced acute lung injury, using GPX4 and SLC7A11 as monitoring markers, and the ferroptosis inhibitor ferrostatin-1 can successfully rescue lung histological changes and has effective therapeutic benefits (112). Puerarin exerts protective effects by reducing the concentration of iron in lung epithelial cells, decreasing the expression and synthesis of GPX4 and GSH, thereby preventing epithelial damage due to ferroptosis (113). Other molecules with similar effects and their mechanisms are also being demonstrated. Electroacupuncture (EA) stimulation at the Zusanli (ST36) inhibits LPS-induced ferroptosis in alveolar epithelial cells by activating α7nAchR, attenuating the lung inflammatory response and thereby alleviating LPS-induced ALI/ARDS (114). Itraconate inhibits macrophage ferroptosis *via* the Nrf2 pathway; hydrogen sulfide attenuates ferroptosis and blocks mTOR signaling in sepsis-induced acute lung injury (115, 116).

#### Sepsis-associated acute renal injury

In various models of acute kidney injury, iron deposition processes have been observed and can be inhibited by ferroptosis inhibitors (e.g. Ferrostatin-1 and lipostatin-1) to exert a renal protective effect (117). Besides the usual occurrence of ferroptosis due to iron overload, mitochondrial-derived reactive oxygen species (ROS) is involved in ferroptosis in some sepsis-related renal injury cells (118). Similarly, inhibition of the mitochondrial-derived antioxidant NADPH oxidase to increase NADPH levels was effective in reducing ferroptosis in renal cells (119). Marin coupling-1 (MCTR1) further suppresses renal tissue ferroptosis and rescues sepsis-related acute kidney injury by regulating Nrf2 expression levels, the effect was also validated by inhibitors of Nrf2 (120).

#### Sepsis-associated encephalopathy

Sepsis-associated encephalopathy (SAE), septic embolism (SEE), and septic metastatic encephalitis (SME) are dynamically occurring neurological complications of sepsis that occur primarily as a result of glutamate-mediated excitotoxic neuronal damage during sepsis-associated uncontrolled neuroinflammation (121). In recent years, treatments targeting ferroptosis have also brought new hope for this neurological complication. In a model of cecum ligation puncture (CLP) sepsis, ferroptosis is progressively observed in the brain with glutathione peroxidase 4 (GPX4) inactivation and upregulation of transferrin. The fer-1 treatment successfully inhibits neuronal ferroptosis and alleviates glutamate excitotoxicity by blocking Xc- system and glutamate receptor N-methyl-D-asperate receptor subunit 2, and these processes ultimately protect synaptic and neuronal integrity during SAE and improve SAE outcomes (122).And ferroptosis inducers will increase sepsis-associated brain injury. Studies by Xuebiao Wei et al. on sepsis-inducing plasmacytoid exosome-derived lncRNANEAT1 suggest that it may promote ferroptosis by modulating the miR-9-5p/transferrin receptor (TFRC) and glutamic-oxaloacetic transaminase 1 (GOT1) axes, ultimately exacerbating SAE (123).

#### Others

Other injuries associated with sepsis include liver injury and immune suppression, and ferroptosis is gradually being identified as its role in these (**Table 1**). Irisin can affect GPX4 expression and attenuate ferroptosis in LPS-treated hepatocytes and CLP sepsis mouse models (124). The effect of ferroptosis on immunosuppression is mainly reflected in reducing the number of immune cells and suppressing immune function. In addition, besides regulating key ferroptosis molecules such as GPX4, GSH, and iron metabolism, targeting ferroptosis regulatory pathways such as Nrf2 and NADPH, the role of other ferroptosis regulatory molecules such as FSP1 and mTOR in reducing sepsis-related organ damage is also a new option for sepsis treatment.

**Table 1:** Current trends in the research of iron death in sepsis.

Sepsis-related organ damage		Experimental models	Detected ferroptosis indicators	Research instruments or molecules	Associated mechanisms	Reference
Sepsis-related cardiac injury	Myocardial injury	lipopolysaccharide (LPS)-induced septic rat/mice LPS stimulated f H9c2 myofibroblasts	prostaglandin endoperoxide synthase 2 (PTGS2), malonaldehyde (MDA) , lipid ROS, mitochondria damage	Ferrostatin-1 (Fer-1), Dexrazoxane (DXZ), erastin, sorafenib, nuclear receptor coactivator 4 (NCOA4) knockdown	nuclear receptor coactivator 4 (NCOA4) mediated- ferritinophagy	98,99,100
	new-onset atrial fibrillation (AF)		PTGS2 ,GPX4, d iron,MDA, Fpn	Fpn knockdown,ferrostatin-1 (Fer-1)	ferroptosis related calcium handling proteins dysregulation	101
Acute lung injury (ALI)/Acute respiratory distress syndrome (ARDS)		LPS treated bronchial epithelial cell line BEAS-2B, LPS induced rat ALI model	malondialdehyde (MDA), 4-hydroxynonenal (4-HNE), iron,SLC7A11 , GPX4,	Ferrostatin-1	Ferrostatin-1 alleviates ALI by inhibiting ferroptosis	103
		LPS induced ALI model in human alveolar epithelial cell A549	cell viability, TNF-α, IL-8, IL-1β,MDA,4-HNE , iron,ROS levels, GPX4,SLC7A11,FTH1,,NOX1,GAPDH	Puerarin, Ferrostatin-1	Puerarin could inhibit the ferroptosis of A549 cells induced by LPS.	104
		lipopolysaccharide (LPS)-induced ARDS mouse model, LPS stimulated MLE-12 cells	Cell Viability ,α7nAchR,MDA,GSH,	Electroacupuncture (EA) stimulation at the Zusanli (ST36)	Activation of α7 nicotinic acetylcholine receptor (α7nAchR)	10
		lipopolysaccharide (LPS)-induced ARDS mouse model	GPX4,PTGS2,MDA,lipid ROS	Itaconate , 4-octyl itaconate (4-OI),ferrostatin-1,	Nrf2 pathways	106
		cecal ligation and puncture (CLP)-induced mouse ALI model	MDA,superoxide dismutase (SOD), glutathione peroxidase (GSH-PX), glutathione reductase activation coefficient (GRAC), iron levels	Hydrogen(GYY4137)	expression of GPx4 and SLC7A11	107
Sepsis-associated acute renal injury (SA-AKI)		lipopolysaccharide (LPS)-induced AKI mouse model LPS-treated human HK-2 cells	MDA,4HNE,GSH	Ferrostatin-1,MitoQ	mitochondria-derived ROS	109
		A septic AKI model in diabetic mice	ASCL4, FTH1, GPX4	NADPH oxidase inhibitor Vas2870,Ferrostatin-1	ROS accumulation	110
		Caecal ligation and puncture (CLP) model,LPS-induced HK-2 cells.	MDA,GSH,non-heme iron	Maresin conjugates in tissue regeneration 1 (MCTR1), Nrf2 inhibitor ML-385,Nrf2 siRNA	Nrf2 signaling.	111
sepsis-associated encephalopathy (SAE)		cecal ligation and puncture (CLP) sepsis model	GPX4,transferrin,MDA	ferrostatin-1 (Fer-1),	glutamate excitotoxicity	113
			ROS, Fe ion, MDA, GSH, GPX4	lncRNANEAT1	miR-9-5p/TFRC and GOT1 Axis	114
sepsis-associated liver injury		cecal ligation and puncture (CLP) sepsis model	GPX4,GSH , lipid peroxidation, iron, mitochondrial morphology	Irisin	GPX4 expression	115

### The potential of ferroptosis-related drugs in the treatment of sepsis

As mentioned above, dexmedetomidine, Irisin, and ferrostatin-1 can act on ferroptosis-related molecules to exert protective effects against septic injury. As clinical studies are conducted, the role of these therapeutic molecules in sepsis treatment should gradually be translated, and the future of ferroptosis in sepsis treatment is promising, and more promising drugs deserve further exploration. In a sepsis model, the protective effect of glutamine (a precursor of GHS synthesis) in acute lung injury can be reversed by BSO (an inhibitor of GSH synthesis), suggesting that it acts through GSH (125). In turn, GSH is an important resistance molecule to ferroptosis, and exogenous supplementation with glutamine to inhibit ferroptosis may be an emerging treatment option for sepsis. Artesunate, widely known for its prominent role in the treatment of malaria, its role in the induction of ferroptosis has only recently been discovered. Subsequent *in vivo* and *in vitro* experiments in liver fibrosis have demonstrated that artemisinin exerts its therapeutic effects by triggering ferritin autophagy, leading to iron accumulation, elevated lipid peroxidation, and reduced antioxidant capacity, and ultimately inducing cellular ferroptosis (126, 127). In the treatment of sepsis, derivatives of artemisinin have shown significant protective effects against sepsis-induced lung injury. Artesunate (AS) upregulates the expression of anti-ferroptosis systems such as Nrf2 and HO-1 and attenuates neutrophil infiltration and pathological damage in lung tissue (128). Similarly, artesunate (AS) has also been shown to activate the mTOR/AKT axis, another regulatory system of ferroptosis, in acute lung injury (129). Whether artemisinin can protect the septic organism by promoting the expression of the antioxidant system, inhibiting iron metabolism-related peroxidation, and ultimately inhibiting the onset of ferroptosis, remains to be investigated. Rapamycin (an mTOR inhibitor) reduces TfR1 expression and subsequently decreases intracellular iron levels, activates the iron regulatory protein-1/2 (IRP1/2) system, and compensates for the downregulation of iron transport proteins to restore iron homeostasis (65). Currently, research on rapamycin in sepsis has been limited to the activation of autophagy and inhibition of pyroptosis through inhibition of mTOR, thereby reducing septic response, septic brain injury, and cognitive impairment in septic mice (130, 131). Given the complex regulation of ferroptosis by mTOR, rapamycin could offer new hope for sepsis by precisely regulating mTOR in sepsis, so that ferroptosis occurs or is restrained appropriately and timely. All in all, research on rapamycin has been long and relatively well established and rapamycin has been put into clinical for a variety of diseases (132). If its protective effect in sepsis through the mechanism of ferroptosis is further investigated and confirmed, then we will have the opportunity to investigate effective drugs for sepsis more expeditiously and safely. In accession to exploring the possibility that drugs with proven clinical applications such as artemisinin and rapamycin exert protective effects in sepsis through ferroptosis, other ferroptosis inhibitors such as punicalagin inhibiting Nrf2 and sulfasalazine affecting the Xc- system and GSH synthesis also showed great therapeutic effects in sepsis have been validated accordingly, but whether their mechanism of action is related to the control of ferroptosis remains to be explored (133, 134).

Although we have discussed various promising drugs as above, especially artemisinin and rapamycin. Nevertheless, research on these drugs is still limited to the experimental stage, and clinical trials are still needed for clinical application. Even if they are put into clinical application, considering the complex condition of sepsis patients and the fragility of their body functions, many issues such as safety, efficacy and resistance, applicability, and economy of these drugs need continuous attention and settlement. In conclusion, the process of exploring the mechanism of ferroptosis-related drugs to clinical application is long and likely to fail at any time, even if the current studies fail, ferroptosis is still a direction full of potential, and the exclusion of a useless drug is the only hope to discover the next possible one. If ferroptosis can be effectively controlled in specific situations and in specific cells without affecting the rest of the organism, it will lead to a breakthrough in the treatment of sepsis. It is no doubt that understanding whether ferroptosis is related to the mechanisms by which these drugs work and exploring newly-development drugs could provide new pathways for sepsis.

## Other perspectives on ferroptosis in sepsis

In summary, the role and possible therapeutic directions of ferroptosis in sepsis and its resulting multiorgan damage and failure are gradually being elucidated, and its future clinical treatment is highly promising. In a cohort of ICU patients, Samya Van collie and her team found that the severity of multiorgan dysfunction and the probability of death in critically ill patients were associated with catalytic iron levels and excessive lipid peroxidation. However, in animal models, blockade of lipid peroxidation by highly soluble iron inhibitor analogues protected mice from injury and death in experimental non-septic multiorgan dysfunction but failed to do the same in septic-induced multiorgan dysfunction (135). The above results may be related to the experimental model, experimental conditions, etc. More validation experiments are worth conducting. Further exploration of the conditions under which ferroptosis occurs in patients with multiple organ failure or sepsis and how to control it for precise and stratified treatment holds the promise of improving clinical outcomes in critically ill patients.

## Conclusion

Sepsis remains a major disease burden due to its high mortality and morbidity. Dysregulation of the host response to infection, causing multi-organ and multi-system damage and dysfunction are its dominant pathogenesis and manifestations. As guidelines for its management are constantly updated, the standard of its treatment and management is improving, but the results are still unsatisfactory. Understanding the underlying mechanisms of sepsis and the development of mechanism-targeted drugs is the most effective approach and the way forward for the treatment of sepsis. Considering the intense metabolic changes in the body in sepsis, ferroptosis, a type of cell death closely related to iron metabolism, lipid metabolism and even some amino acid metabolism, has attracted lots of attention. In this review, we describe the signaling pathways, participating molecules and other regulatory molecules involved in the development of ferroptosis, with focuses on the interaction of these signaling pathways and regulatory molecules, their roles in sepsis, and the potential pharmacological effects of targeting them. However, there are still some limitations in this study. We have tried our best to collect and review the relevant literature, but some mechanisms and regulatory relationships are still not fully described. Due to the current research progress, the discussion of ferroptosis-related organ damage in this paper mostly focuses on the pharmacology or the involvement of ferroptosis, and the specific mechanism mediated by it has not been explored in detail, but this may provide ideas for future research and encourage researchers to explore in this direction.

Ferroptosis is a relatively new area, and its role in the pathophysiological process and treatment of sepsis remains in the exploration stage. Intervention in the development and progression of ferroptosis through multiple drugs and pathways is undoubtedly a new direction for future research in the treatment of sepsis.

## Author contributions

CN contributed to the conception and guidance of the manuscript. XL wrote the manuscript, generated figures and tables. ZG and GR helped to review and edited the manuscript. All authors contributed to the article and approved the submitted version.

## Funding

The work was supported by National Natural Science Foundation of China (No. 82072226), Beijing Municipal Science and Technology Commission (No. Z201100005520049)

## Acknowledgments

I would like to thank all the tutors and fellow students who have encouraged and helped in the completion of this article. Thanks for their contributions!

## Conflict of interest

The authors declare that the research was conducted in the absence of any commercial or financial relationships that could be construed as a potential conflict of interest.

## Publisher’s note

All claims expressed in this article are solely those of the authors and do not necessarily represent those of their affiliated organizations, or those of the publisher, the editors and the reviewers. Any product that may be evaluated in this article, or claim that may be made by its manufacturer, is not guaranteed or endorsed by the publisher.
